# Progress and promise

**DOI:** 10.7554/eLife.44799

**Published:** 2019-01-23

**Authors:** Randy Schekman

**Keywords:** eLife, scientific publishing, peer review, open access, research assessment

## Abstract

As he prepares to step down as the Editor-in-Chief of eLife, **Randy Schekman** reflects on the origins of the journal, the eLife approach to peer review, and current challenges in scientific publishing.

In December 2010 the Howard Hughes Medical Institute (HHMI), the Max Planck Society and the Wellcome Trust invited leading figures in the life and biomedical sciences to a meeting at the HHMI Janelia Research Campus to consider the creation of a new open-access journal that would publish outstanding research in these fields. Many of those present expressed impatience with the delays and difficulties they had experienced in publishing what they considered to be excellent work in the pages of three journals: Cell, Nature and Science.

Mark Walport, the director of the Wellcome Trust at the time, memorably remarked that "the process of science peer review needs to be owned by professional scientists" in order to curtail the "endless iterations of nit-picking" that were common during peer review at the most selective journals (Jump, 2011). The consensus was that the business plans of these journals were based on exclusivity, overseen by the teams of professional editors who made all the decisions about manuscripts at these journals, and an overreliance on the Journal Impact Factor.

The situation was particularly toxic for early-career researchers, who felt that they had to publish in one of these journals in order to secure a permanent research position (Schekman, 2013). Although questions were asked about the need for yet another journal, there was support for the idea of a selective journal where working scientists, rather than professional editors, would take all the editorial decisions.

At the time of the meeting I was coming to the end of a five-year term as Editor-in-Chief of the Proceedings of the National Academy of Sciences (PNAS), and had been prepared to stay for a second term. However, Robert Tjian, my colleague at UC Berkeley and president of HHMI at the time, urged me to consider leading this new effort. I was intrigued by the possibility of this new venture, particularly given its solid financial foundation and the opportunity of starting afresh. Harold Varmus, then director of the National Cancer Institute, advised that a new journal would have to do something different in order to distinguish it from other journals in the evolving publishing landscape. Thus, when offered the position, I accepted with enthusiasm and took Varmus’s sage advice to "think different" to heart.

## Setting up eLife

My first steps were to appoint two Deputy Editors (Fiona Watt and Detlef Weigel), a team of Senior Editors who were all active scientists in the major areas of the life sciences, and a manager for the editorial office (Mark Patterson). At a meeting in New York in December 2011 we agreed on a peer-review process for the journal. All new submissions would be assigned to a Senior Editor, who would decide (often in consultation with a member of our soon-to-be established Board of Reviewing Editors) whether or not to invite a full submission that would be sent to referees for full peer review: this initial decision is taken by professional editors at the most selective journals.

Thinking about ways to improve the peer-review process for manuscripts that survive this initial stage, I recalled how editorial staff at PNAS were able to consult with one another online as submissions moved through the peer-review process, so we agreed that eLife editors and reviewers should discuss a manuscript and their reports on it before reaching a consensus decision. This consultation – which is overseen by the Reviewing Editor who is handling the manuscript – has become a central element of the eLife peer-review process: once the last report has been received, each reviewer receives an email asking them to read and comment on the other reports (which include the name of each reviewer). If the reviewers broadly support publication, they agree on the revisions that need to be made in order for the manuscript to be accepted. This is a significant change to the standard approach because the reviewers, in collaboration with the editor, focus on identifying the most important revisions for the author. In addition, the consultation allows the reviewers to learn from each other and, if necessary, to revise their views on a manuscript in the light of comments from reviewers who might know more about a particular topic or technique.

One of my goals had been to make the peer-review process more open and constructive: over the years I felt that peer review had become somewhat toxic and destructive, with reviewers taking advantage of the veil of anonymity to make more and more unnecessarily negative comments about the papers they were reviewing. In the eLife process reviewers know that they will have to defend their views in an open discussion with the other reviewers before an editorial decision is rendered on a manuscript, and I am convinced that this has fostered a more positive approach to peer review, and am pleased to see that a number of other journals have experimented with similar approaches (King, 2017).

One of my goals had been to make the peer-review process more open and constructive

It is worth emphasizing three other points about the eLife approach to peer review: i) in the case of a positive decision the individual referee reports are normally not sent to the authors: rather authors are sent a detailed decision letter that contains a list of the essential revisions agreed by the Reviewing Editor and the reviewers during the consultation (such as requests for extra experimental data or further analysis) and a list of minor revisions; ii) extra experiments are only requested if they are essential to support the major claims in the manuscript and can be completed within two months; iii) in most cases the revised manuscript is assessed by just the Reviewing Editor, rather than sending it back to all the reviewers: this, combined with our practice of only requesting essential revisions, helps to speed up the publication process.

Other important aspects of the eLife peer-review process include: there is no target acceptance rate for eLife – editors accept all the papers that reach the standard we expect (by contrast, the acceptance rates of the most selective journals are still largely constrained by page budgets); there is no limit on the length of papers – authors can have as many words and figures as they need; and the decision letter and the authors' response to this letter are published with the paper, along with the names of those referees who are happy for their identities to be made public. Unlike the most selective journals, eLife is not in the business of selling magazines or subscriptions. Instead, our editors are selected for their expertise, and they are encouraged to accept for publication those papers that combine true scholarship and responsible behavior, rather than identifying papers on the latest fads that may help to increase our impact factor. We have opposed the use of the impact factor since day one because we feel it is meaningless, particularly when it is used to assess individual papers or scientists (and it is regrettable that so many scientists seem to be in thrall to this number).

## Launch and rapid growth

By the time we opened for submissions in June 2012 we had 15 Senior Editors (excluding Detlef, Fiona and myself) and 175 Reviewing Editors, and by the end of 2012 we had published 27 Research Articles. Fast forward to the present and we received 7671 submissions in 2018 and published over 1250 research papers (in the form of Research Articles, Short Reports, Research Advances and Tools and Resources papers). We now have over 340 Reviewing Editors, 43 Senior Editors and three Deputy Editors (Eve Marder became a Deputy Editor in 2015, and Anna Akhmanova replaced Fiona Watt in 2018).

We are also committed to improving both the gender and geographical balance among our Editors: this is important to us because an analysis of all submissions to eLife between 2012 and 2017 found that "women and authors from nations outside of North America and Europe were underrepresented both as gatekeepers (editors and peer reviewers) and last authors" (Murray et al., 2018). Sadly, this conclusion is consistent with what has been found at other journals (see, for example, Helmer et al., 2017; Lerback and Hanson, 2017).

We have opposed the use of the impact factor since day one because we feel it is meaningless, particularly when it is used to assess individual papers or scientists.

Given the generous support we received from our funders, we did not charge a publication fee until the end of 2016. However, as we knew we would eventually have to go it alone, we introduced a publication fee of $2500 per paper in January 2017. The fee was set at this level to cover our marginal publication costs, with the funds we receive from HHMI, the Max Planck Society, Wellcome and the Knut and Alice Wallenberg Foundation largely being used to support fixed publishing costs and the development of our journal infrastructure (please see Setting a fee for publication for further details).

In addition to peer review, I am pleased that eLife has been innovative in other ways. For example, we established an Early-Career Advisory Group in 2014 to act as a "voice for early-career researchers within eLife", appointed an early-career researcher to our Board of Directors in January 2018, and have launched several initiatives to increase the number of early-career researchers who review manuscripts for the journal. In collaboration with the Center for Open Science, we are publishing the results of the Reproducibility Project: Cancer Biology, an initiative to explore the reproducibility of preclinical research in cancer biology, and to identify the factors that influence reproducibility more generally. We have also worked with other organizations on a variety of projects, such as the DORA campaign "to improve the ways in which the outputs of scholarly research are evaluated". And many eLife papers have a plain-language summary (called a digest) that explains the content and context of the paper for the general reader (King et al., 2017).

Although open-access journals continue to increase their share of the market for research papers, progress has been slower than hoped for as the large commercial publishers and some scientific societies with substantial publishing operations have resisted, usually to protect their substantial profit margins (which go to shareholders at commercial publishers or are used to fund activities unrelated to publishing at societies). Fortunately, the ground is now shifting rapidly, most notably with a far-reaching proposal in Europe called Plan S. A variety of other organizations, including my own beloved libraries of the University of California, are also pushing for change. The overall goal is to oblige journals that rely on a subscription model, and more importantly the commercial journals that extract their profit from the free labor provided by academics, to find a satisfactory business plan to survive the open access future.

Another positive development in scientific publishing over the past few years has been the growth of preprint servers in the life and biomedical sciences, notably bioRxiv, which allow researchers to make their results available as quickly and as widely as possible. A number of journals had been exploring new approaches to scientific publishing before the launch of eLife, notably journals published by BMC, BMJ, EMBO and PLOS, and this spirit of innovation has continued with these publishers and others: examples include the development of the Open Research publishing platform by F1000Research (Rodgers, 2018), and an increase in the number of journals publishing referee reports and other information about the peer-review process (Polka et al., 2018). And at eLife we are currently analyzing the results of a trial in which, for papers that are sent for peer review, authors decide how to respond to the issues raised by the reviewers (Patterson and Schekman, 2018).

Looking ahead, I see several challenges and opportunities for eLife. One is for the journal to continue to grow and to continue to advocate for change in peer review and scientific publishing more generally, both individually and as part of organizations such as DORA. Another is to continue to build an end-to-end open-source platform for scientific publishing. We have started to build a submission and peer-review platform in collaboration with a number of other like-minded organizations, and are also in the process of adapting our publishing platform so that it can be used by other journals and publishers. The hope is that this platform could be adopted by society publishers and other non-profits to reduce their costs, and thus reduce their reliance on the income from journal subscriptions. I see this as a crucial development as it will help societies and non-profits survive in an environment that is dominated by the large commercial publishers and societies with substantial publishing operations.

## A personal note

Although I am enormously gratified by the success and future promise of eLife, I decided to step down for personal reasons. My wife of 44 years died in September 2017 of complications from Parkinson’s Disease. At the time of her death, I had been in casual conversation with a representative of the Sergey Brin Family Foundation, which has generously supported research on Parkinson’s Disease. When my wife died, I was asked by the Brin Foundation to chair an effort to identify and support key elements of basic research focused on understanding the origin(s) and mechanism(s) of disease progression, and I am now helping to build an international network of Parkinson’s Disease geneticists, cell and molecular biologists, neuroscientists and physicians to tackle this scourge. Our goal is to advance our basic understanding as an essential prelude to the development of new drugs and surgical procedures. I am eager to take on this challenge and am grateful to the Brin Foundation for helping to turn my personal grief into something constructive.
